# Clay Sculpture‐Inspired 3D Printed Microcage Module Using Bioadhesion Assembly for Specific‐Shaped Tissue Vascularization and Regeneration

**DOI:** 10.1002/advs.202308381

**Published:** 2024-03-06

**Authors:** Huimin Fang, Jingyi Ju, Lifeng Chen, Muran Zhou, Guo Zhang, Jinfei Hou, Wenbin Jiang, Zhenxing Wang, Jiaming Sun

**Affiliations:** ^1^ Department of Plastic Surgery Union Hospital Tongji Medical College Huazhong University of Science and Technology Wuhan 430022 China

**Keywords:** 3D bioprinting, 3D printing, assembly, bioadhesion, hydrogel, tissue engineering

## Abstract

3D bioprinting techniques have enabled the fabrication of irregular large‐sized tissue engineering scaffolds. However, complicated customized designs increase the medical burden. Meanwhile, the integrated printing process hinders the cellular uniform distribution and local angiogenesis. A novel approach is introduced to the construction of sizable tissue engineering grafts by employing hydrogel 3D printing for modular bioadhesion assembly, and a poly (ethylene glycol) diacrylate (PEGDA)‐gelatin‐dopamine (PGD) hydrogel, photosensitive and adhesive, enabling fine microcage module fabrication via DLP 3D printing is developed. The PGD hydrogel printed micocages are flexible, allowing various shapes and cell/tissue fillings for repairing diverse irregular tissue defects. In vivo experiments demonstrate robust vascularization and superior graft survival in nude mice. This assembly strategy based on scalable 3D printed hydrogel microcage module could simplify the construction of tissue with large volume and complex components, offering promise for diverse large tissue defect repairs.

## Graphical abstract

Inspired by traditional Chinese clay sculptures, a revolutionizing tissue repair strategy with 3D‐printed hydrogel microcages for irregular defects is proposed. In vivo experiments have shown that the hydrogel modules can be well vascularized and that the grafts placed in the hydrogel module can survive better in vivo than subcutaneously transplanted.

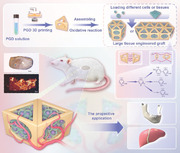

## 1 Introduction

Tissue engineering and regenerative medicine are advancing fields aiming to create in vitro tissue‐engineered grafts for in vivo tissue repair.^[^ ^1^, ^2^ ^]^ However, large in vitro grafts often suffer from an inadequate nutrient supply and necrosis due to poor vascularization.^[^ ^3^, ^4^ ^]^ Microtissue assembly methods enhance graft cellularity and survival but face challenges, such as in vivo assembly difficulties and displacement.^[^ ^5^ ^]^ 3D printing technology is based on 3D models designed on‐demand, and models with fine structures are manufactured through software‐based layered discretization and numerical control molding. Broadly speaking, 3D printing that directly serves the biomedical field can be referred to as 3D bioprinting. It typically involves manipulating bioinks containing cells to construct active structures. 3D bioprinting technology offers certain advantages over other strategies in the construction of human tissues. However, there are also certain limitations to consider.^[^ ^6^, ^7^, ^8^ ^]^ In the case of individualized tissue defects, such as tumors and trauma, the repair of customized tissue defects requires many complex steps, such as scanning, reverse modeling, and printing, to construct grafts to fit irregular defects.^[^ ^9^, ^10^ ^]^ In clinical translation applications, this prolongs the intraoperative waiting time for patients after anesthesia. Therefore, established in vivo tissue engineering grafting strategies face many challenges with respect to clinical translation.

The tissue engineering chamber is an in vivo surgical device developed using stiff materials (e.g., plastic or silicon) that provide a relatively isolated environment for graft tissues or cells.^[^ ^11^, ^12^ ^]^ The main theory involves introducing a wall structure that acts as a tissue compartment and improves graft survival in vivo by reducing the direct infiltration of inflammatory cells, thereby promoting vascularization and reducing the compression of the surrounding tissue. The tissue‐engineered chamber model has been successfully used for in vivo transplantation of a variety of tissues and cells, including cardiac muscle, adipose, islet, and liver cells.^[^ ^13^, ^14^, ^15^, ^16^ ^]^ The 3D‐printed microcage strategy was derived from a tissue engineering chamber model. Tissue engineering microcages are similar to tissue engineering chambers, with each microcage unit having a precise LEGO structure loaded with cells or tissues. In clinical translational applications, the principle of modular stacking proves invaluable when swiftly and flexibly crafting grafts of varying shapes to mend specific tissue defects.^[^ ^17^, ^18^ ^]^ Compared with traditional tissue engineering transplantation strategies, microcage modules offer several advantages. First, pre‐produced 3D printed microcage modules can be easily constructed into different shapes through assembly, and these can be directly used to repair specific tissue defects during surgery. This approach, involving reverse modeling and subsequent 3D printing of scaffolds, surpasses traditional methods in terms of speed and flexibility, eliminating intraoperative delays. Second, microcage modules mitigate tissue compression due to their shell‐like structure, leading to higher in vivo tissue engineering graft survival rates than those for direct in vivo grafting. Third, the microcage module strategy enables the easy on‐demand arrangement of grafts with different cells, making it possible to construct tissue modules with multiple cell types and vascularized microenvironments.

Since biological tissues are typically soft, the microcage modules, initially modeled after the LEGO concept using solid materials, have certain limitations in terms of applicability. With good biocompatibility and adjustable hardness, hydrogels may be more suitable materials for tissue engineering microcage modules. However, limited research has focused on how to assemble hydrogels modularly. Traditional Chinese clay sculptures often involve the use of soft clay to shape partial modules first, followed by module assembly using the adhesive properties of the clay. Inspired by traditional Chinese clay sculptures, this study proposes a strategy in which different modules are 3D printed in advance and assembled using adhesives to construct large‐volume models.

Bio‐adhesion is a common phenomenon in nature, and its principles are widely used in the development of biomaterials.^[^ ^19^, ^20^ ^]^ Among numerous adhesion molecules employed in biomaterial development, mussel adhesion proteins, rich in catechol groups, stand out owing to their capacity to adhere to a wide range of chemical and physical bonds.^[^ ^21^ ^]^ Many studies have focused on modifying conventional biomaterials with catechol groups, enabling bio‐adhesion through catechol group oxidation or metal chelation reactions.^[^ ^22^, ^23^ ^]^ By utilizing catechol‐based modified hydrogels in the creation of microcage modules, their adhesive properties can be harnessed to facilitate the assembly of microcages and the integration of grafts with recipient tissue. Furthermore, the development of hydrogel 3D printing technology offers new ideas for biomanufacturing.

By choosing hydrogels with temperature‐sensitive or light‐sensitive properties and enabling controlled layer‐by‐layer curing of the hydrogel precursor solution under the guidance of a digital model, high‐precision hydrogel models can be produced on demand using 3D bioprinting.^[^ ^24^, ^25^ ^]^ Tissue‐engineered microcage modules prepared using hydrogels with 3D bioprinting technology are expected to improve graft survival better in vivo due to the better biocompatibility of the hydrogel.^[^ ^26^, ^27^ ^]^ Currently, there are three main bioprinting printing methods: extrusion‐based bioprinting, droplet‐based bioprinting or inkjet, and laser‐assisted bioprinting. Extrusion bioprinting involves the use of nozzle extrusion to linearize biological ink, creating a thin layer structure through continuous linear structure; several thin layers are then stacked to form a 3D structure. This printing method has relatively limited printing speed and accuracy.^[^ ^28^ ^]^ Droplet jet bioprinting uses a nozzle to spray droplets to print fluid materials; however, its printing accuracy and thickness have certain limitations.^[^ ^29^, ^30^ ^]^ Laser‐assisted bioprinting uses a bio‐ink that can be cured during laser projection and photocured layer‐by‐layer to form a 3D structure. Its printing accuracy is high, the printing speed is fast, and the development is rapid in recent years. Digital light projection 3D printing technology is a widely used type of laser‐assisted bioprinting.^[^ ^31^, ^32^ ^]^ However, a hydrogel material that simultaneously exhibits adhesion, 3D printability, and biocompatibility has not yet been reported.

**Figure 1:**
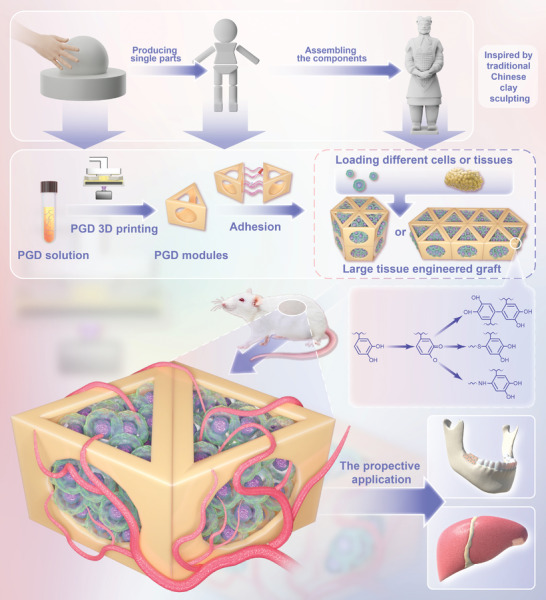
Illustration of hydrogel 3D printing for large grafts.

In this study, we introduce a novel poly(ethylene glycol) diacrylate (PEGDA)‐gelatin‐dopamine (PGD) composite hydrogel material and propose a scalable strategy for tissue engineering grafts using microcage modules based on this hydrogel. We designed and prepared a PGD composite hydrogel material that is both photosensitive for high‐precision 3D printing and bioadhesive for stable adhesion between the hydrogel and biological tissue after photocuring. The composite hydrogel material was subsequently used to prepare microcage modules using DLP 3D printing technology. In vivo experiments showed that these microcage modules are biocompatible, and blood vessels can grow into the microcage along the 3D printed pores, providing an in vivo vascularized microenvironment for the transplanted tissue inside the microcage. Furthermore, in vivo tissue‐engineered bone graft subcutaneous transplantation experiments demonstrated that the use of hydrogel microcage modules improved the survival rate of bone grafts. These results underscore the potential of the bio‐adhesive, photosensitive 3D‐printed hydrogel microcage module‐based strategy, which is scalable and readily assemblable, in the field of tissue engineering (**Figure** **1**). This innovative approach holds the promise for addressing the current limitations in tissue engineering research, particularly with regards to the development of large‐volume grafts in vitro and achieving high graft survival rates in vivo. Consequently, this study provides a viable solution for the clinical translation of tissue engineering and regenerative medicine research.

## 2 Results

### 2.1 Synthesis of PGD Hydrogels

**Figure 2:**
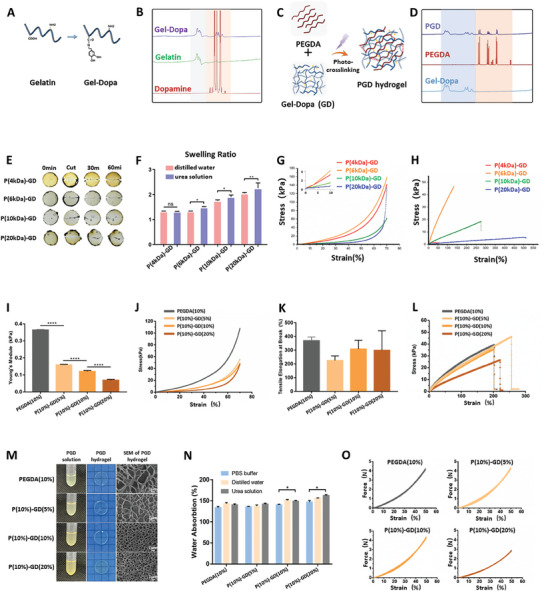
Synthesis of PGD hydrogels. A) Dopamine was grafted onto gelatin macromolecules via a crosslinking reaction using EDC/NHS to synthesize GD molecules. B) The NMR hydrogen spectra showed that GD exhibited the characteristic peaks of both gelatin and dopamine. C) The prepared GD was mixed with PEGDA and subsequently photo‐crosslinked to form a PGD hydrogel. D) NMR analysis indicated that PGD displayed characteristic peaks of both PEGDA and GD. E) The higher the molecular weight of PEGDA, the stronger are the hydrogen bonding and self‐healing capabilities of the PGD hydrogel. F) The smaller the molecular weight of PEGDA, the denser the crosslinked network inside the hydrogel, and the lesser the swelling. G,H) The strain–stress curve of the PGD hydrogels indicated that a higher molecular weight of PEGDA resulted in a lower Young's modulus and a softer hydrogel. I,J) Young's modulus and compression stress–strain curve analyses revealed that a higher concentration of GD resulted in a softer PGD hydrogel. K,L) The elongation rupture rate and tensile stress–strain curve analysis indicated that the addition of GD did not significantly affect the brittleness of the K–L hydrogel. M) The porosity of the PGD composite hydrogels gradually decreased with increasing GD concentration. N) The greater water absorption observed in the urea solution indicates the formation of hydrogen bonds. O) Fatigue tests demonstrated that the PGD composite hydrogel exhibited good elasticity.

To graft the catechol group of dopamine hydrochloride onto the gelatin molecular chain a crosslinking reaction was initiated between dopamine hydrochloride and gelatin, catalyzed by 1‐(3‐dimethylaminopropyl)−3‐ethylcarbodiimide hydrochloride (EDC) and N‐hydroxysuccinimide (NHS). The amino group on dopamine hydrochloride reacted with the carboxyl group on gelatin to form an amide bond (**Figure** **2**A), resulting in the formation of gelatin‐dopamine (GD) (Figure S1A, Supporting Information). nuclear magnetic resonance (NMR) hydrogen spectroscopy (Figure 2B) and FTIR spectroscopy (Figure S1B, Supporting Information) confirmed that GD possessed the characteristic peaks of both gelatin and dopamine hydrochloride. Subsequently, GD was mixed PEGDA to form a homogeneous aqueous solution, which was crosslinked using UV light to form PEGDA‐gelatin‐dopamine (PGD) composite hydrogels (Figure 2C). NMR hydrogen spectroscopy results showed that the PGD composite hydrogel possessed the characteristic peaks of both PEGDA and GD (Figure 2D).

PEGDA is the generic term used to describe a group of compounds. The smaller molecular weight of PEGDA leads to a higher proportion of olefinic bonds, which makes it easier to form hydrogels through photo‐crosslinking. However, these hydrogels may be brittle and easily breakable. To screen for a suitable molecular weight of PEGDA, we prepared composite hydrogels using 4, 6, 10, and 20 kDa PEGDA, and GD in a ratio of 10% mass concentration of PEGDA to 20% mass concentration of GD. The synthesized PGD composite hydrogels were denoted as P(4 kDa)GD, P(6 kDa)GD, P(10 kDa)GD, and P(20 kDa)GD. These hydrogels were prepared in a disk shape and cut in the middle to observe their self‐healing abilities. It was found that the higher the molecular weight of PEGDA in the hydrogel, the more pronounced its self‐healing capability (Figure 2E). The swelling ratio was determined using distilled water and urea solution, and it was found that the swelling of the composite hydrogel increased with increasing molecular weight of PEGDA in the hydrogel. Furthermore, a statistical difference was observed in the swelling of the hydrogel in distilled water and urea solutions when the molecular weights of PEGDA were 6, 10, and 20 kDa (Figure 2F). The increased swelling in urea solution indicated significant hydrogen bond formation within the hydrogel, as these bonds disassembled in the presence of urea. To evaluate the mechanical properties of the hydrogels, cylindrical hydrogels with a diameter of 9 mm and a height of 9 mm were prepared using the mold method and tested for compressive and tensile stress‐strain. The higher molecular weight of PEGDA led to a lower slope of the compressive strain–stress curve, indicating a lower Young's modulus and a softer hydrogel (Figure 2G). Moreover, the higher molecular weight of PEGDA led to a greater tensile length of the hydrogel in the tensile stress–strain curve, indicating a more elastic hydrogel (Figure 2H). Based on the above findings, we chose 10 kDa PEGDA for the synthesis of PGD composite hydrogels in subsequent experiments.

The addition of GD resulted in a slight decrease in the hardness of the composite hydrogel (Figure 2I–J), whereas the elongation fracture rate did not show a significant change (Figure 2K,L). The porosity of the PGD composite hydrogels gradually decreased with increasing GD concentration (Figure 2M). Additionally, the water absorption of the hydrogel increased after the addition of GD and was significantly higher in the 8% urea solution than in the PBS solution, especially at higher GD concentrations (Figure 2N). The hydrogels exhibited favorable elasticity, as demonstrated by fatigue tests (Figure 2O).

### 2.2 3D Printing Properties of PGD Hydrogels

**Figure 3:**
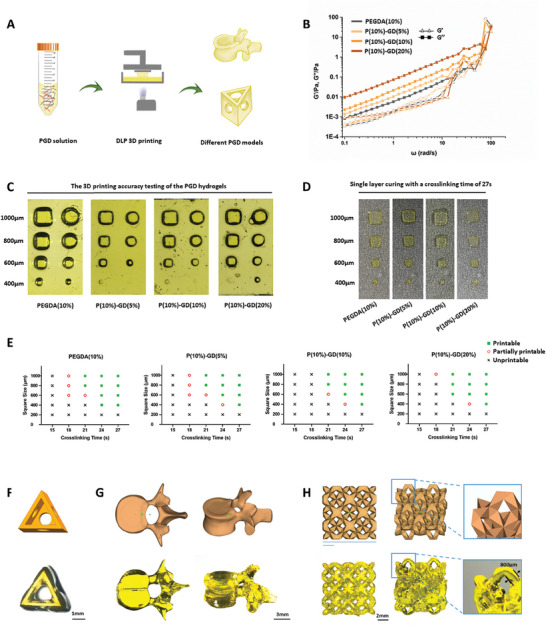
A) 3D printing properties of PGD hydrogels. Schematic of the procedure for 3D printing of PGD hydrogel. B) Rheological tests of the PGD solution showed that, as the GD concentration increased, the PGD solution became viscous. C) The 3D printing accuracy testing of the PGD hydrogels with different concentrations was examined, and different PGD solutions can print models with an accuracy of 600 µm. D,E) The GD concentration did not affect the printing accuracy of PGD hydrogels. F–H) PGD hydrogels can be used to prepare various models with fine structures using DLP 3D printing.

Highly accurate PGD hydrogel models were prepared using a DLP 3D printer (**Figure** **3**A). The energy storage modulus *G*' and loss modulus *G*″ of the PGD composite hydrogel precursor solution were measured using a rheometer. As the GD concentration increased, the loss modulus of the hydrogel precursor solution increased, indicating that the liquid became viscous (Figure 3B). In this study, square and cylindrical models with varying accuracies (1000, 800, 600, and 400 µm) and a height of 1 mm were designed. We assessed the printing performance of the PGD composite hydrogels with different concentrations using a DLP 3D printer. PEGDA alone possessed high printing accuracy, and the addition of GD enabled the printing of 600 µm models (Figure 3C). Importantly, the addition of GD essentially did not affect the photo‐crosslinking time of the PGD composite hydrogel (Figure 3D,E and Figure S2, Supporting Information). This suggests that a wide range of models with fine structures can be printed on a DPL 3D printer using PGD composite hydrogels (Figure 3F–H).

### 2.3 Adhesion of PGD Hydrogels

**Figure 4:**
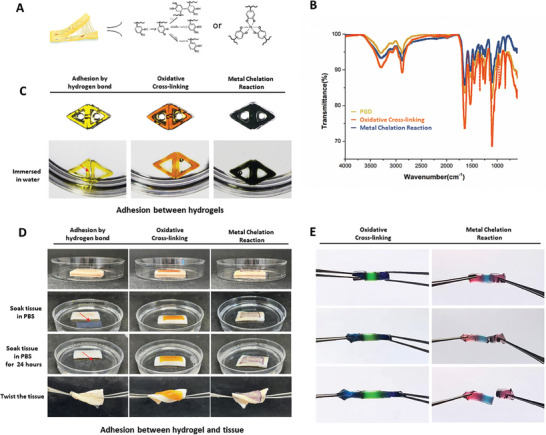
Adhesion of PGD hydrogels. A) The principle of PGD hydrogel adhesion is illustrated in panel. B) Fourier‐transform infrared spectroscopy revealed that the bond energy of the oxidation reaction of the PGD hydrogel was higher. C) The PGD hydrogels that adhered via oxidative and metal chelation reactions maintained stable crosslinking in water. D) The PGD composite hydrogel that adhered to the tissue was also able to maintain stable adhesion after immersion in PBS solution for 1 d after the oxidative and metal chelation reactions. E) The oxidatively crosslinked hydrogels remained stably adhered at the crosslinking reaction interface when stretched to twice their length, whereas the metal‐crosslinked hydrogels broke from the adhesion interface.

PGD hydrogels can adhere to various materials via hydrogen bonds (Figure S3A, Supporting Information). Rheological tests on different PGD hydrogels indicated that PEGDA likely provided reversible hydrogen bonding at the hydrogel interface (Figure S3B, Supporting Information). Adhesion in PGD hydrogels primarily relies on chemical bonding principles, including oxidative reactions and metal chelation (**Figure** **4**A). To compare the bonding mode and bond energy strength of PGD composite hydrogels crosslinked either oxidatively by sodium periodate or by metal using ferric chloride, we conducted Fourier infrared spectroscopy on the reacted hydrogels. The results showed that the bond energy of the chemical bond formed by oxidative crosslinking was higher than that of the chemical bond formed after metal crosslinking (Figure 4B). In subsequent studies, hydrogels were assembled and adhered via oxidative reactions and metal crosslinking. The PGD composite hydrogels maintained stable crosslinking in water after adhesion (Figure 4C). The PGD composite hydrogel that adhered to the tissue was also able to maintain stable adhesion after immersion in PBS solution for 1 d (Figure 4D). To further illustrate the difference, cylindrical PGD hydrogels were prepared using the mold method, stained with water‐soluble dyes, and then subjected to tension testing after crosslinking at the interface using sodium periodate and ferric chloride solutions. The oxidatively crosslinked hydrogels remained stably adhered when stretched, while the metal‐crosslinked hydrogels separated from the adhesion interface (Figure 4E).

**Figure 5:**
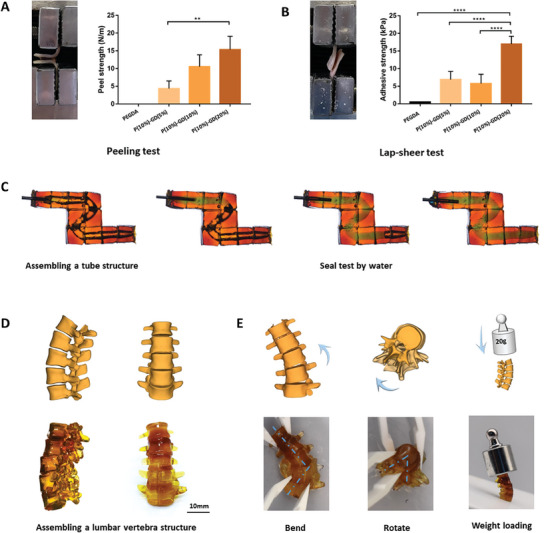
Adhesion of PGD hydrogel models. A,B) Lap shear test and peel test showed that the adhesion strength of the hydrogels gradually increased with increasing concentration of GD. C) A 3D‐printed hydrogel with pore channels was assembled to construct a hollow pipe structure without leakage in the sealing test. D,E) The PGD hydrogel spines prepared by DLP 3D printing were assembled, and the models could bend and rotate like a normal human spine and could also withstand pressure.

A sodium periodate solution was added dropwise to the surface of two pieces of pigskin, and the PGD hydrogel was placed between them for adhesion. The adhesive strength of the hydrogel after the oxidative reaction with pigskin was examined using lap shear and peel tests. The results revealed that PEGDA alone did not exhibit adhesion, and the adhesion of the hydrogel gradually increased with increasing concentrations of GD. PGD‐1, PGD‐2, and PGD‐3 contained 5%, 10%, and 20% GD, respectively. In the lap shear test, the adhesion strengths of PGD‐1, PGD‐2, and PGD‐3 were 6.875 ± 2.316, 5.775 ± 2.623, and 16.98 ± 2.192 kPa, respectively (**Figure** **5**A). In the peel test, the adhesion strengths of PGD‐1, PGD‐2, and PGD‐3 were 4.37 ± 2.113, 10.53 ± 3.366, and 15.34 ± 3.762 kPa, respectively (Figure 5B). The results showed that the adhesion of the PGD hydrogels to the pigskin surface was mediated by the reaction between the catechol groups and the hydroxyl or sulfhydryl groups on the skin. PGD‐3, which contained a higher concentration of catechol‐based gelatin, exhibited a significantly higher adhesion strength than the other two groups, and the differences were statistically significant. Using sodium periodate, a 3D‐printed hydrogel with pore channels was assembled to construct a hollow pipe structure, and the liquid was injected into the pipe. The results showed that the liquid passed smoothly through the pipe without leaking from the adhesive interface (Figure 5C). PGD hydrogels can be 3D‐printed into individual spine models, which can be assembled via the oxidative reaction of the catechol moiety (Figure 5D). The assembled spine models were bent and rotated to a maximum bending angle of approximately 60° and a maximum rotation angle of approximately 40°. Moreover, they could withstand compression up to 136 times their own weight (hydrogel mass 147 mg, weight mass 20 g) (Figure 5E).

### 2.4 Biocompatibility of PGD Hydrogels

**Figure 6:**
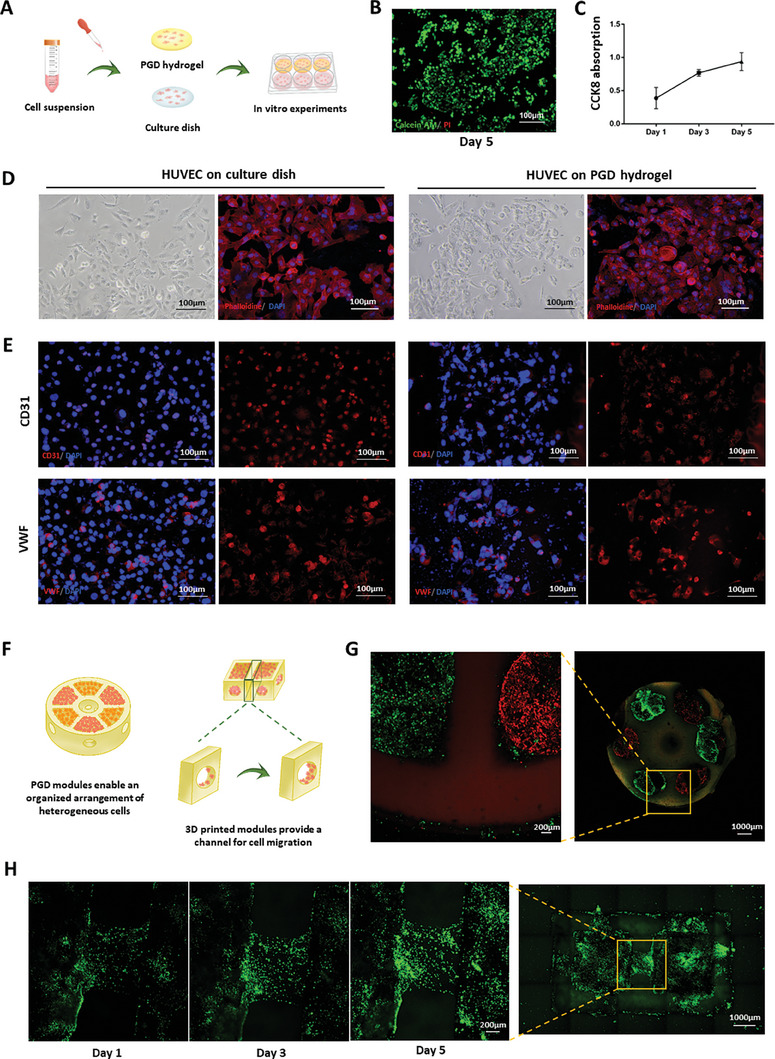
Biocompatibility of PGD hydrogels. A) Schematic of the in vitro biocompatibility test. B,C) The MTT assay and live/dead cell staining results indicated that the HUVECs inoculated on the hydrogel surface exhibited excellent cell viability. D) The cellular morphology of HUVECs grown on the hydrogel was slightly different from that of cells grown on flat dishes. E) Immunofluorescence staining revealed that the protein expression of HUVECs remained unchanged when cultured on the hydrogel. F) Cell spheres were placed within the 3D‐printed PGD hydrogel modules, and the microcage strategy enabled the organized arrangement of heterogeneous cells and provided a channel for cell migration. G) In a 3D‐printed PGD hydrogel model, GFP‐HUVECs and Cherry‐HUVECs were arranged in different regions on demand, and cells were observed crossing the reserved channels. H) Cells grew along the channel of the PGD hydrogel module.

Disks of PGD hydrogels with different molecular weights of PEGDA were prepared using the mold method, and GFP‐human umbilical vein endothelial cells (HUVECs) were cultured on the surface of the hydrogels to observe cell proliferation (**Figure** **6**A). Light microscopy and laser confocal microscopy showed that GFP‐HUVECs adhered to the PGD composite hydrogel surface, proliferating and growing (Figure S4A, Supporting Information). Notably, when the molecular weight of PEGDA was 20 kDa, the morphology of GFP‐HUVECs became significantly rounded. Therefore, we chose 10 kDa molecular weight PEGDA to synthesize the PGD composite hydrogels in subsequent experiments. HUVECs were seeded on the surface of the PGD composite hydrogels with different concentrations of GD, and the number of cells that adhered to the surface of the hydrogels was observed under a light microscope after 3 d. As the concentration of GD increased, more cells adhered to the hydrogel surface (Figure S4B, Supporting Information). The HUVECs were seeded onto the surface of the PGD composite hydrogel and stained with calcein AM and propidium iodide (Figure 6B). The CCK8 cell proliferation assay results showed a gradual increase in the number of cells over time (Figure 6C). HUVECs were then seeded onto the surfaces of glass slips and PGD composite hydrogels, and cell morphology was observed using light and laser confocal microscopy, with fluorescein isothiocyanate (FITC)‐phalloidin staining being performed to visualize the cytoskeleton. The results revealed the rounded morphology of the cells grown on the hydrogel surface (Figure 6D). Immunofluorescence staining for CD31 and von Willebrand Factor (VWF) was performed to assess whether HUVECs could express common proteins when cultured on the hydrogel surface. The results demonstrated that the HUVECs on the PGD composite hydrogel surface expressed CD31 and VWF normally (Figure 6E). The cell spheres were placed within the 3D‐printed PGD hydrogel modules, which enabled an organized arrangement of heterogeneous cells and provided channels for cell migration (Figure 6F). In the 3D‐printed PGD hydrogel model, GFP‐ and Cherry‐HUVECs were arranged in different regions on demand, and cells were observed crossing the channels reserved for printing (Figure 6G). GFP‐HUVECs migrated and proliferated along the channels reserved for 3D printing on the hydrogel, as observed through dynamic monitoring (Figure 6H).

PEGDA and PGD hydrogels were subcutaneously embedded in the backs of the rats, and a fibrous capsule on the surface of the hydrogels was observed at 2 and 12 weeks (Figure S4C, Supporting Information). PCR analysis was conducted at 3 and 14 d to assess the expression of IL‐1, IL‐10, and TNF‐α, revealing no significant differences compared to the control group (Figure S4D, Supporting Information). Furthermore, HE staining of the heart, lungs, liver, kidneys, and spleen was performed four weeks after the subcutaneous embedding of PGD to evaluate any potential adverse effects. No significant differences were observed compared to the control group (Figure S5, Supporting Information).

### 2.5 Vascularization within PGD Hydrogel Microcage Modules

**Figure 7:**
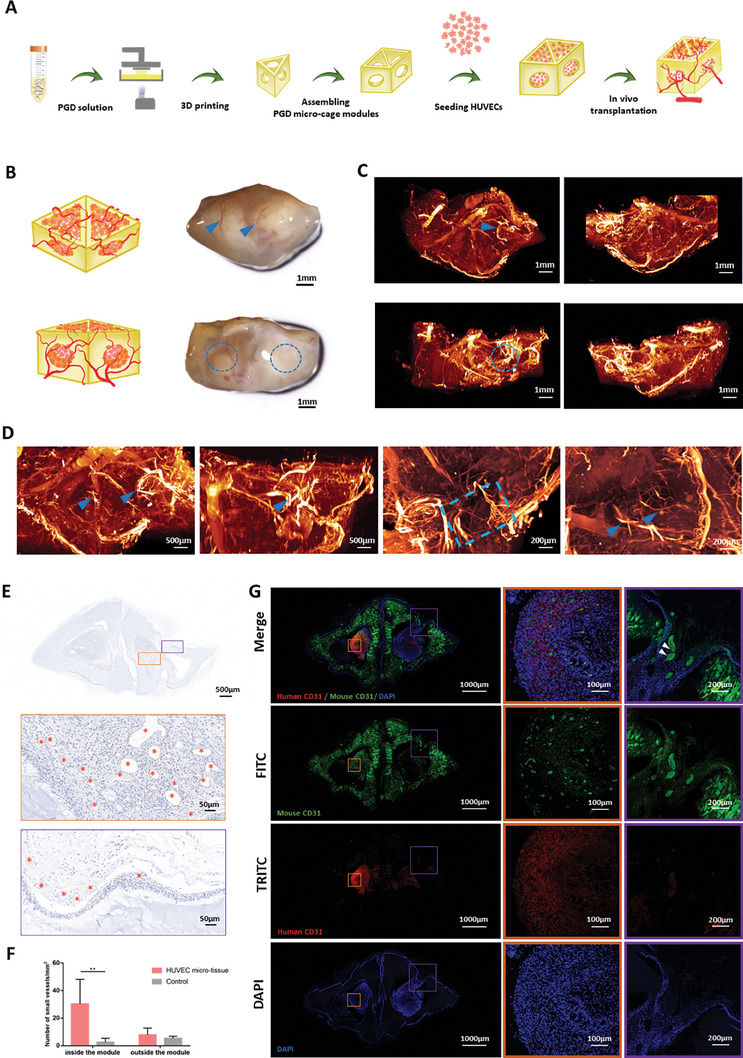
Vascularization within PGD hydrogel microcage modules. A) Schematic of the in vivo transplantation of HUVEC‐loaded PGD hydrogel microcage modules. B) Angiogenesis was observed from the fibrous capsule to the interior of the PGD microcage modules (blue arrows). C,D) 3D scanning and reconstruction of CD31‐labeled mouse vessels indicated that they formed a well‐vascularized microenvironment in the PGD microcage modules. E) CD31 immunohistochemical staining of graft sections showing the formation of many vascular structures inside the PGD module. F) With the loading of HUVECs, the number of vessels inside the PGD microcage modules was significantly higher, indicating that the PGD microcage modules could be well‐vascularized. Using mouse CD31 immunofluorescence staining, we observed that murine blood vessels grew into the PGD microcage modules along the 3D‐printed channel (white arrow). G) Additionally, human CD31 immunofluorescence staining demonstrated the survival of HUVECs within the PGD microcage modules.

The HUVEC‐loaded microspheres were placed inside the 3D‐printed PGD hydrogel microcage modules and subsequently transplanted subcutaneously into nude mice (**Figure** **7**A). After 4 weeks, the grafts were harvested, and blood vessels from the fibrous capsule grew into the graft through the hole on the surface of the hydrogel (Figure 7B). By scanning the sample in three dimensions and labeling the vessels with a mouse CD31 antibody, we reconstructed the graft, revealing numerous vascular structures both outside and inside the PGD hydrogel microcage modules (Figure 7C). The vasculature grew into the PGD hydrogel microcages through the printed hole (Figure 7D). Immunohistochemical staining of sample sections using a mouse CD31 antibody showed the presence of CD31‐positive circumferential vascular structures in the inner and outer fibrous envelopes (Figure 7E). A PGD hydrogel microcage module without HUVECs was used as a control. Five sections were randomly selected from different slices, and the number of blood vessels (with a diameter greater than 20 µm) in the grafts of both groups was counted. The results indicated the successful vascularization of the PGD microcage modules in vivo (Figure 7F). To investigate the origin of the vessels within the hydrogel module and assess the survival of the transplanted HUVECs, immunofluorescence staining using human CD31/mouse CD31/ DAPI was performed. The results showed the extensive survival of the transplanted HUVECs within the module, as indicated by the red fluorescence of human CD31. The vessels within the graft expressed green fluorescence of mouse CD31 and exhibited growth along the 3D printed pores (Figure 7G).

### 2.6 Application of PGD Hydrogel Modules

**Figure 8:**
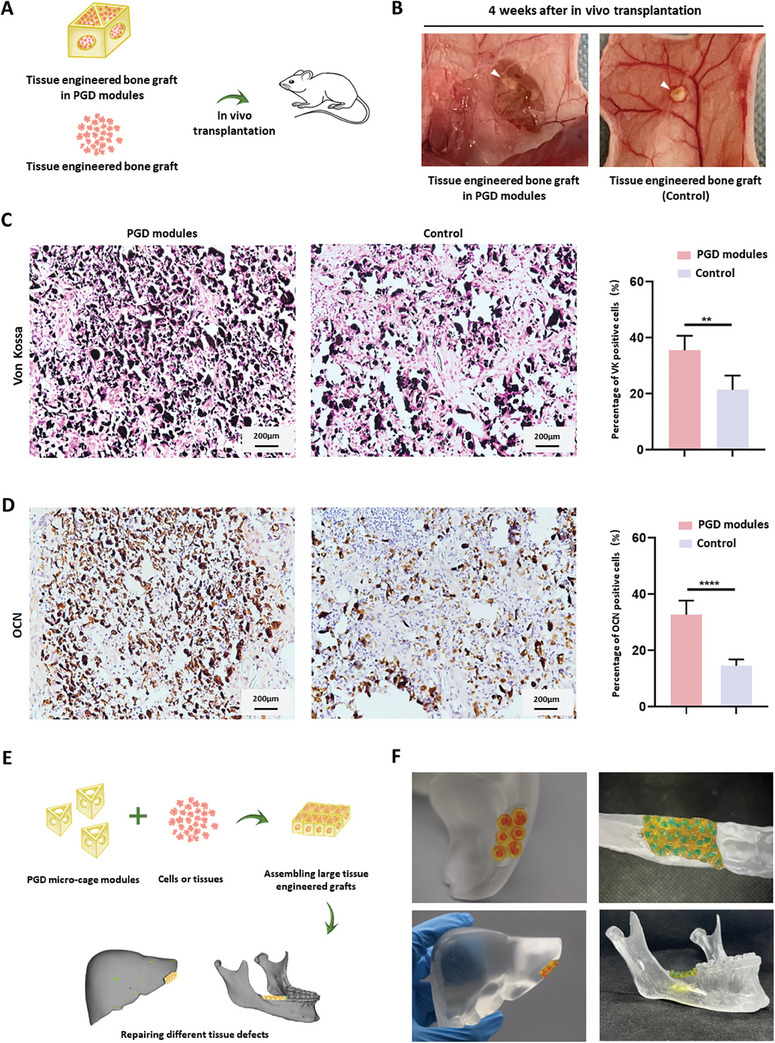
Application of PGD hydrogel modules. A,B) Tissue‐engineered bone grafts constructed in vitro were loaded onto PGD microcage modules and transplanted subcutaneously into nude mice. C,D) Von Kossa and OCN immunohistochemical staining of the grafts cultured after 4 weeks showed that C, D) more osteogenically differentiated cells survived in the PGD microcage modules, and the area ratio of Von Kossa‐positive and OCN‐positive cells in the PGD microcage modules were significantly higher than those in the control group. E,F) PGD microcage modules loaded with cells and tissues can be assembled to construct large‐volume grafts that are expected to repair various tissue defects.

Tissue‐engineered bone grafts were created by the osteogenic induction of BMSC‐loaded microspheres and placed into 3D‐printed PGD hydrogel microcage modules. These constructs were transplanted subcutaneously into nude mice, whereas the control group received tissue‐engineered bone grafts placed directly subcutaneously in nude mice (**Figure** **8**A). Grafts in the control group exhibited a significant reduction in size, whereas those in the PGD hydrogel microcage modules were protected by the cage structure and retained a greater volume of calcified structures in vivo (Figure 8B). Von Kossa staining and OCN (osteocalcin) immunohistochemical staining demonstrated better survival of the bone grafts in the PGD hydrogel microcage modules than those in the control group (Figure 8C,D). Five areas were randomly selected from Von Kossa and OCN immunohistochemical slices, and the proportion of positive cells was determined. The results showed that Von Kossa‐positive cells in the PGD microcage modules accounted for about 35.5 ± 5.25% of the total area, significantly higher than the 21.44 ± 5.02% in the control group (*p* < 0.01) (Figure 8C). Similarly, the proportion of OCN‐positive cells in the PGD microcage modules was approximately 32.7 ± 5.005% of the total area, significantly higher than the 14.57 ± 2.270% in the control group (*p* < 0.0001). These results indicate that the cells in the PGD microcage modules survived better (Figure 8D). INOS/ CD163 double‐labelling immunofluorescence staining, which refers to M1/M2 Macrophages, confirmed that there were fewer M1/M2 macrophages infiltrated in grafts in the PGD hydrogel modules (Figure S6, Supporting Information).

The PGD hydrogel microcage modules can accommodate microtissues containing different cell types, enabling the construction of large‐volume grafts. Through adhesion of the hydrogel and subsequent integration with the tissue defect site, precise repair of tissue defects using tissue‐engineered grafts is possible. This strategy offers a promising approach for the precise repair of tissue defects and formation of tissue‐engineered grafts (Figure 8E,F).

## 3 Discussion

In the fields of tissue engineering and regenerative medicine, the construction of large irregular grafts with improved in vivo survival rates presents a significant challenge. The use of tissue‐engineered microcage modules for the extended assembly of large grafts has emerged as a promising solution. Traditional Chinese clay sculptures can include various shapes with intricate structures. Soft, adhesive clay is used to knead local modules, which are then assembled using adhesion to form a whole. Inspired by this construction method, we propose a strategy in which soft hydrogel 3D printing is used to prepare tissue engineering microcage modules, followed by assembly using a hydrogel adhesive to build large‐volume grafts. There is a need for customized materials to meet specific research requirements in this area. Therefore, we developed a novel composite hydrogel material, PGD, with unique properties suitable for photosensitive 3D printing and adhesion. The PGD hydrogel was successfully 3D printed into microcage modules. These modules can be assembled and expanded, exhibit excellent vascularization in vivo, and promote the survival of tissue‐engineered grafts when transplanted in vivo. This study demonstrates that the in vivo construction strategy of tissue‐engineered grafts utilizing bio‐adhesive 3D printed hydrogel microcage modules has distinct advantages. This represents a new and promising solution for the clinical translation of tissue engineering research and provides a pathway for more efficient and successful clinical applications in the field.

The development of bioadhesive hydrogels has garnered significant attention in recent years. A commonly used adhesion principle was inspired by mussels. Mussel adhesion proteins possess abundant catechol groups, which enable adhesion via various chemical and physical bonds.^[^ ^33^, ^34^ ^]^ Many studies have focused on modifying hydrogel materials with catechol groups.^[^ ^35^, ^36^ ^]^ Hydrogels incorporating composite catechol groups have a wide range of applications, including in skin wound repair, hemostasis, biosensors, and wearable smart devices.^[^ ^37^, ^38^, ^39^ ^]^ In this study, we prepared a composite hydrogel material, PGD, leveraging the adhesion principle of catechol moieties found in mussel proteins. This adhesion was achieved through the reaction of catechol groups after 3D printing and molding. Initially, GD was prepared and mixed with PEGDA to form a solution to prepare the PGD composite hydrogel. This PGD hydrogel serves as a double‐network composite hydrogel in which PEGDA molecules confer photosensitivity.^[^ ^40^, ^41^ ^]^ PEGDA forms a separate network through photo‐crosslinking, whereas GD molecules are interspersed within the PEGDA network via hydrogen bonding. Upon photo‐crosslinking, adhesion between hydrogels or between hydrogels and tissues can be achieved using sodium periodate solutions. The adhesion properties of the PGD composite hydrogel mainly stem from the catechol moiety, as evidenced by the stronger hydrogel adhesion at higher GD molecule concentrations.

Hydrogels are highly suitable as tissue engineering scaffolds because they have a high‐water content and adjustable hardness, making them compatible with biological systems. Thus, in recent years, research on 3D printed hydrogel tissue engineering scaffolds has gained significant attention.^[^ ^42^, ^43^ ^]^ To fabricate these scaffolds, modified natural biomacromolecules, such as methacrylate‐based gelatin (GelMA), methacrylate‐based hyaluronic acid (HAMA), and methacrylate‐based collagen, are widely used.^[^ ^44^, ^45^, ^46^ ^]^ However, natural biomacromolecular hydrogels often have limited mechanical properties. Consequently, the development of composite hydrogel materials tailored to specific research needs has become a major focus. PEGDA, a biologically inert and photosensitive hydrogel, is commonly used in the construction of composite hydrogels.^[^ ^47^, ^48^, ^49^ ^]^ Composite hydrogels with enhanced properties were obtained by combining PEGDA with biomacromolecules. The newly developed PGD composite hydrogel exhibited excellent photosensitivity, plasticity, and biocompatibility, similar to those of PEGDA. Moreover, it can be 3D printed with fine structural precision using DLP 3D printing technology.

The development of hydrogel 3D printing technology has enabled the construction of large volumes of irregular tissue‐engineered grafts. However, in clinical applications, it is difficult to construct large‐volume grafts adapted to the shape of the defective tissue in the face of acute tissue injury owing to the cycle of cell inoculation, culture, and induction required for tissue engineering graft construction in a short time.^[^ ^50^, ^51^ ^]^ Furthermore, 3D printing tissue‐engineered scaffolds adapted to irregular defects require additional steps, such as scanning, reverse modeling, and sterile 3D printing. These additional steps can contribute to longer intraoperative anesthesia times.^[^ ^52^ ^]^ Consequently, repairing irregular large‐volume tissue defects is a major clinical challenge and a research bottleneck in the field of regenerative medicine. To address this challenge, tissue‐engineered microtissue construction strategies and tissue‐engineered modular assembly strategies provide potential solutions.^[^ ^53^, ^54^ ^]^ Subbiah et al. proposed a microcage construction strategy for tissue engineering. First, microcage modules are 3D printed and then filled with tissue‐engineered microtissues for in vivo transplantation.^[^ ^17^ ^]^ In this study, we used DLP 3D printing technology to prepare PGD composite hydrogels for different microcage modules and subsequently filled them with microtissues to construct irregular large‐volume grafts. In vivo experiments demonstrated significantly higher bone tissue survival within the microcage modules than that in the control group, which underwent direct subcutaneous grafting. This result may be attributed to the supportive effect of the microcages, which alleviates mechanical compression of the graft by the surrounding tissue, leading to a significant improvement in graft survival compared to that for direct grafting. In addition to its supportive role, the PGD hydrogel microcage module provides a certain level of isolation, reducing the infiltration of inflammatory cells into the graft, thereby improving the graft survival rate in vivo.

In tissue engineering studies, poor vascularization of the implantation site is often a problem during the in vivo transplantation phase, and post‐transplantation tissues may have poor survival rates due to a lack of nutrient supply. Methods to improve blood supply to the recipient area, which can significantly improve the survival rate of tissue‐engineered grafts in vivo, have been evaluated.^[^ ^55^, ^56^ ^]^ Sun et al. showed that the addition of exosomes and growth factors with angiogenic properties improves the survival rate of grafts by promoting angiogenesis.^[^ ^57^, ^58^ ^]^ In some studies, graft survival was improved by transplanting vascular loops or vascular‐like organs into the graft area.^[^ ^59^ ^]^ These studies suggest that the in vivo survival rate of grafts can be improved by angiogenesis of grafts in vivo. Previous studies have confirmed that additional endothelial cells can form new blood vessels and connect with tissue vessels in situ.^[^ ^60^ ^]^ In this study, the PGD hydrogel microcage module was well‐vascularized in vivo after the addition of HUVEC‐laden microtissues. For in vivo applications, the distribution of transplanted tissues within the microcage modules can be predesigned such that the HUVEC microtissues are evenly distributed in the graft, thus inducing the formation of vascular networks within the grafts and increasing the survival rate in vivo. This strategy provides a new approach for in vivo tissue engineering transplantation studies. In future studies, we may develop hydrogels loaded with molecules for different tissues and conduct in situ defect repair in vivo.

This study had some limitations. First, it cannot be denied that the mechanical properties of the PGD hydrogel must be modified depending on the tissue type. In addition, the in vivo inflammatory response and degradation time should be studied further to achieve clinical implantation standards. In addition, loading different types of microtissues into PGD hydrogel microcage modules to construct large‐volume tissue‐engineered grafts for preclinical trials in large animals, such as pigs and rhesus monkeys, would be more effective for confirming the feasibility of this tissue engineering strategy.

## 4 Conclusion

In conclusion, drawing inspiration from Chinese clay sculpture assembly, we introduce a novel approach to construct sizable tissue engineering grafts. We successfully designed and synthesized a biocompatible PGD hydrogel with excellent 3D printing capability and post‐printing adhesion properties. The hydrogel can be 3D printed into microcage modules with precise pore structures, and these modules can be assembled and expanded through adhesion. Importantly, the microcage modules facilitated vascularization, leading to improved survival rates of the tissue‐engineered grafts in vivo. Moving forward, microcage modules can be easily assembled to create large‐volume tissue‐engineered scaffolds of various shapes that can be filled with different types of grafts. In specific regions, HUVEC‐loaded microtissues can be incorporated to enhance vascularization and further improve graft survival rates in vivo. This scalable assembly strategy based on 3D printed hydrogel microcage modules holds great potential for future applications in the fields of biomaterials and regenerative medicine.

## 5 Experimental Section

### Preparation of PGD Hydrogel

To prepare GD, 2 g of gelatin (G7041, Sigma‐Aldrich, USA) was dissolved in 100 mL of PBS buffer. Next, 0.5 g of EDC (E7750, Sigma‐Aldrich, USA) and 0.3 g of NHS (130672, Sigma‐Aldrich, USA) were added to the solution, and the pH was adjusted to 5 using hydrochloric acid solution. The solution was stirred for 30 min to activate it. Subsequently, 1 g of dopamine (H8502, Sigma‐Aldrich, USA) was dissolved in 5 mL of deionized water, and the dopamine solution was added dropwise to the reaction solution. The mixed solution was shaken overnight at 20–25 °C (150 rpm). On day 2, the solution was dialyzed using a 3500 Da dialysis bag for 48 h, with at least six fluid changes. On day 4, the dialysate was pre‐lyophilized at ‐20 °C and then freeze‐dried for 48 h to obtain GD.

For the preparation of PGD hydrogels, a 0.1% light absorber solution was created by dissolving 10 mg of lemon yellow (T0388, Sigma‐Aldrich, USA). Then, 100 mg of PEGDA (031109, MeloPEG, China) was added to 1 mL of light absorber solution and mixed thoroughly. Additionally, 200 mg of GD and 5 mg of photo initiator lithium phenyl (2,4,6‐trimethylbenzoyl) phosphinate (LAP) (900889, Sigma‐Aldrich, USA) were added to the solution. A small amount of dilute hydrochloric acid was added to adjust the pH until the catechol gelatin was fully dissolved. The solution was added to molds of different shapes and irradiated with 365 nm UV light to obtain PGD hydrogels.

### Characterization of PGD Hydrogels

To analyze the composition of GD molecular and PGD composite hydrogels, NMR hydrogen spectroscopy (Bruker 600 MHz, Germany) was used. A composite material sample (20 mg) was dissolved in heavy water and placed in a nuclear magnetic tube after being fully dissolved. An NMR hydrogen spectrometer (Bruker, 600 MHz, Germany) was employed for detection, and the data were processed using MestReNova software. The chemical bonding structure of the GD molecules was determined using Fourier infrared spectroscopy (Thermo Scientific Nicolet 6700, USA). Appropriate quantities of GD, gelatin, and dopamine were prepared separately. Samples were mixed and ground using potassium bromide crystals for detection. The FTIR measurements were conducted in the frequency range of 4000 to 400 cm^−1^, and the data were processed using Origin software. Different concentrations of PGD solutions were placed in a rheometer (Haake Mars60, Germany), and the modulus test (oscillation mode) was performed under the frequency test with fixed strain in the frequency range of 0.1 to 100 rad s^−1^. The experimental data were processed using Origin software. Different concentrations of PGD hydrogels were prepared in cylindrical and flake shapes using the mold method and placed in the fixture of a tensile testing machine (ZHIQU ZQ770, China). The samples were tightly fixed using two clamps and stretched or compressed at a constant rate of 10 mm min^−1^. The results of the stress–strain curves, Young's modulus, and cyclic compression experiments were plotted using Origin software. Different concentrations of PGD composite hydrogels were prepared as flat sheets using the mold method and immersed in different solutions to fully absorb water. The diameters and weights were measured before and after the process. Additionally, swelling ratio and water absorption were calculated. The morphology of the PGD hydrogels was observed using a cold‐field‐emission scanning electron microscope (S‐4800, HITACHI, Japan) fitted with a low‐temperature sample carrier (Quorum PP3000T). To prepare for observation, the hydrogels were initially frozen in liquid nitrogen and then transferred to the sample carrier chamber, where the temperature was raised to ‐90 °C and subsequently cooled to ‐180 °C to sublimate the solid water. Prior to observation, the surfaces of the hydrogels were coated with platinum using a sputter coater.

### 3D Printing of PGD Hydrogels

UG NX 10.0 software was used to design the print model and generate the STL file. In the DLP 3D printer (EFL, model 8601, China), the printing parameters were set as follows: a layer height of 20 µm, light intensity of 14 mW cm^−2^, 10 base layers, base layer exposure time of 26 s, sheet exposure time of 24 s, *Z* speed of 25 mm min^−1^, peeling distance of 1 mm, peeling speed of 10 mm min^−1^, and peeling recovery speed of 100 mm min^−1^. The printed model was sliced, and the configured solution was added dropwise to the printer tank. After 3D printing, the models were taken out and placed in an EP tube and sealed at 4 °C.

### Adhesion of PGD Hydrogels

A solution containing 5 mg mL^−1^ sodium periodate (311448, Sigma‐Aldrich, USA) and 16.3 mg mL^−1^ ferric chloride (157740, Sigma‐Aldrich) was prepared. Sodium periodate solution was added dropwise to the hydrogel adhesion interface and then pressed for 10 s to allow the hydrogel to fully react and achieve oxidative crosslinking adhesion. An iron chloride solution was added dropwise to the hydrogel adhesion interface and pressed for 10 s to allow the hydrogel to fully react and achieve crosslinked metal adhesion.

The subcutaneous fat was removed from pig skin obtained from the market. The pigskin was then cut into 1 cm × 2 cm rectangles, and the surface grease was wiped off with alcohol. Next, 10 µL of sodium periodate solution or iron chloride solution was added drop by drop onto the surface of the pigskin. The PGD hydrogel was gently placed on the pigskin's surface and pressed to ensure the hydrogel fully reacted with the pigskin's surface. The adhesion strength test was conducted in a tensile machine (ZHIQU ZQ770, China).

### Biocompatibility and Vascularization of PGD Hydrogels

Sterilized hydrogels were placed in 24‐well plates and HUVECs were digested, centrifuged, and resuspended to prepare a cell suspension of 5 × 10^6^ cells mL^−1^. Then, 50 µL of cell suspension was added dropwise to the surface of each hydrogel and incubated in an incubator for 2 h. Subsequently, 1 mL of culture medium was added to each well and the solution was changed on alternate days. Live and dead cells were stained at different time points, and cell survival was observed using a laser confocal microscope (Nikon, Ni‐E, Japan). CCK8 (Beyotime, C0037, China) assays were performed to evaluate cell proliferation. FITC‐Phalloidin cytoskeleton staining (Solarbio, CA1620, China), vWF (Servicebio GB11020, China), and CD31 (Servicebio, GB113151, China) immunofluorescence staining were performed, and the differences in cell morphology and protein expression were observed under laser confocal microscopy on the hydrogels compared to those in flat culture. Microtissues containing GFP‐HUVECs and RFP‐HUVECs were transplanted into specially designed PGD hydrogel microcage modules and cultured in a 5% CO_2_ incubator set at 37 °C, with fluid changes on alternate days. Migration of HUVECs into the channels of the hydrogel was observed under a laser confocal microscope (Nikon, Tokyo, Japan) at different time points.

Microtissues containing HUVEC were transplanted into sodium periodate‐oxidized crosslinked PGD hydrogel microcage modules and subsequently transplanted under the dorsal skin of nude rats. The grafts were cultured for 4 weeks. Immunohistochemical staining for CD31 and immunofluorescence staining for human‐derived CD31 (Thermo Fisher, 14‐0319‐82, USA) and murine‐derived CD31 (Thermo Fisher, MA5‐38125, USA) were performed to evaluate HUVEC survival and angiogenesis in the grafts. 3D imaging of solvent‐cleared organs was used to observe angiogenesis in the PGD microcage modules. Blood vessels in the tissues were stained with a CD31 antibody. Subsequently, the tissues were made transparent using the FDISCO kit (Jarvisbio, China), and the tissues were then imaged in three dimensions using a light sheet transparency imaging system (LiTone XL, Light innovation Technology, China), with a depth imaging distance of 5 µm.^[^ ^61^ ^]^

The hydrogels were embedded under the skin on the back of 250 g male SD rats and taken at different time points for HE staining and immunohistochemical staining, as well as at different time points for PCR detection.

### Application of PGD Hydrogel Microcage Modules

The steps for the construction of tissue‐engineered bone microtissues were the same as those described previously.^[^ ^5^ ^]^ A sodium periodate solution was added to assemble the PGD hydrogel microcage modules. Osteogenesis‐induced microtissues were loaded into microcage modules. An incision was made in the middle of the back of nude mice and subcutaneously separated on both sides. The microtissues were then subcutaneously placed on the left side of the back of nude mice, which were then loaded with microtissues on the right side of the back. The grafts were harvested to observe cell survival at different time points.

The Von Kossa staining procedure was carried out as follows: the specimen underwent dehydration using an ethanol gradient, embedded in paraffin, and sectioned into slices. Sections were then stained with 2% silver nitrate and gently rinsed with 5% Na_2_SO_4_ for 1–2 min. Subsequently, counterstaining was performed using alkaline magenta for 10 s. The stained slices were then sealed and areas of tissue with calcification, as indicated by black staining, were observed under a microscope.

Immunohistochemical staining of osteocalcin was carried out as follows: the specimen was dehydrated using an ethanol gradient, embedded in paraffin, and sectioned into slices. Next, the slices were incubated with rabbit anti‐rat osteocalcin primary antibody overnight at 4 °C. Subsequently, the sections were incubated with a goat anti‐rabbit secondary antibody and developed using diaminobenzidine tetrahydrochloride. Finally, the slides were sealed, and the positively stained dark brown cells were visualized under a microscope.

The experimental animals were kept, operated on, and treated according to the requirements of the Ethics Committee of Tongji Medical College of Huazhong University of Science and Technology. The animals were housed and managed in the Laboratory Animal Center of Tongji Medical College.

### Statistical Analysis

Each assay was performed with a minimum of three replicates, and the quantitative results were analyzed using GraphPad Prism (version 8.0). Differences were considered statistically significant when *p*‐values were below 0.05 (*p* < 0.05).

## Conflict of Interest

The authors declare no conflict of interest.

## Supporting information

Supporting Information

Supplemental Video 1

## Data Availability

The data that support the findings of this study are available from the corresponding author upon reasonable request.
