# Supplementary material for: Clay Sculpture‐Inspired 3D Printed Microcage Module Using Bioadhesion Assembly for Specific‐Shaped Tissue Vascularization and Regeneration
Source: Adv Sci (Weinh). 2024 Mar 6;11(21):2308381. doi: 10.1002/advs.202308381 (PMC11151015; doi:10.1002/advs.202308381)
Supplement: Supplementary file 1 — Supporting Information [file ADVS-11-2308381-s001.pdf]

ADVANCED SCIENCE
Open Access

Supporting Information

for *Adv. Sci.*, DOI 10.1002/advs.202308381

Clay Sculpture-Inspired 3D Printed Microcage Module Using Bioadhesion Assembly for Specific-Shaped Tissue Vascularization and Regeneration

Huimin Fang, Jingyi Ju, Lifeng Chen, Muran Zhou, Guo Zhang, Jinfei Hou, Wenbin Jiang, Zhenxing Wang and Jiaming Sun**

Supporting Information

Clay sculpture-inspired 3D printed microcage module using bio-adhesion assembly for specific-shaped tissue vascularization and regeneration

Huimin Fang, Jingyi Ju, Lifeng Chen, Muran Zhou, Guo Zhang, Jinfei Hou, Wenbin Jiang, Zhenxing Wang, Jiaming Sun**

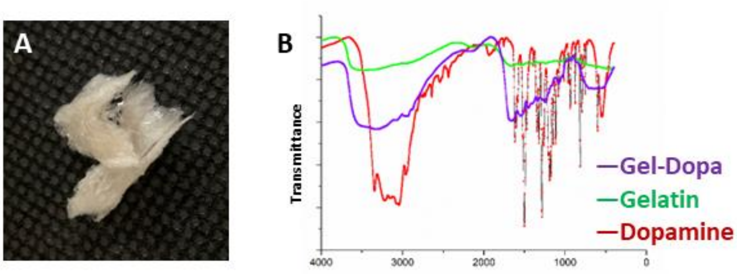

Figure S1. GD (A) and its Fourier transform infrared spectroscopy (B).

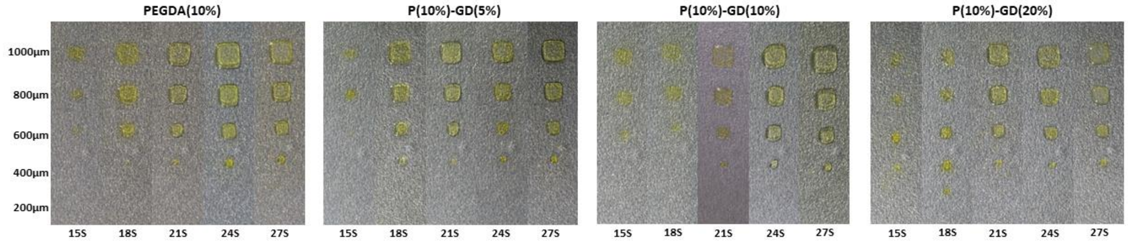

Figure S2. Printability of PGD under different crosslinking times when exposed to square patterns with side lengths ranging from 200-1000μm. The addition of GD essentially did not affect the photo-crosslinking time of the PGD composite hydrogel.

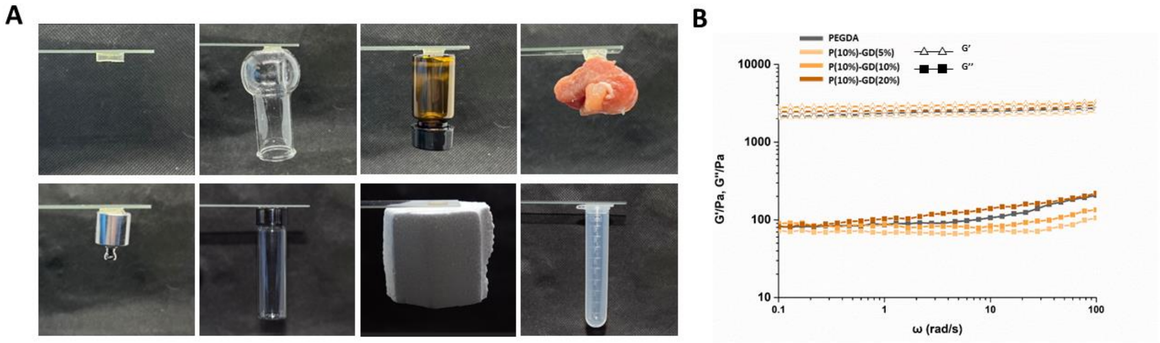

Figure S3. PGD hydrogels can adhere to a variety of different materials by hydrogen bonding(A); Rheological tests of different PGD hydrogels(B).

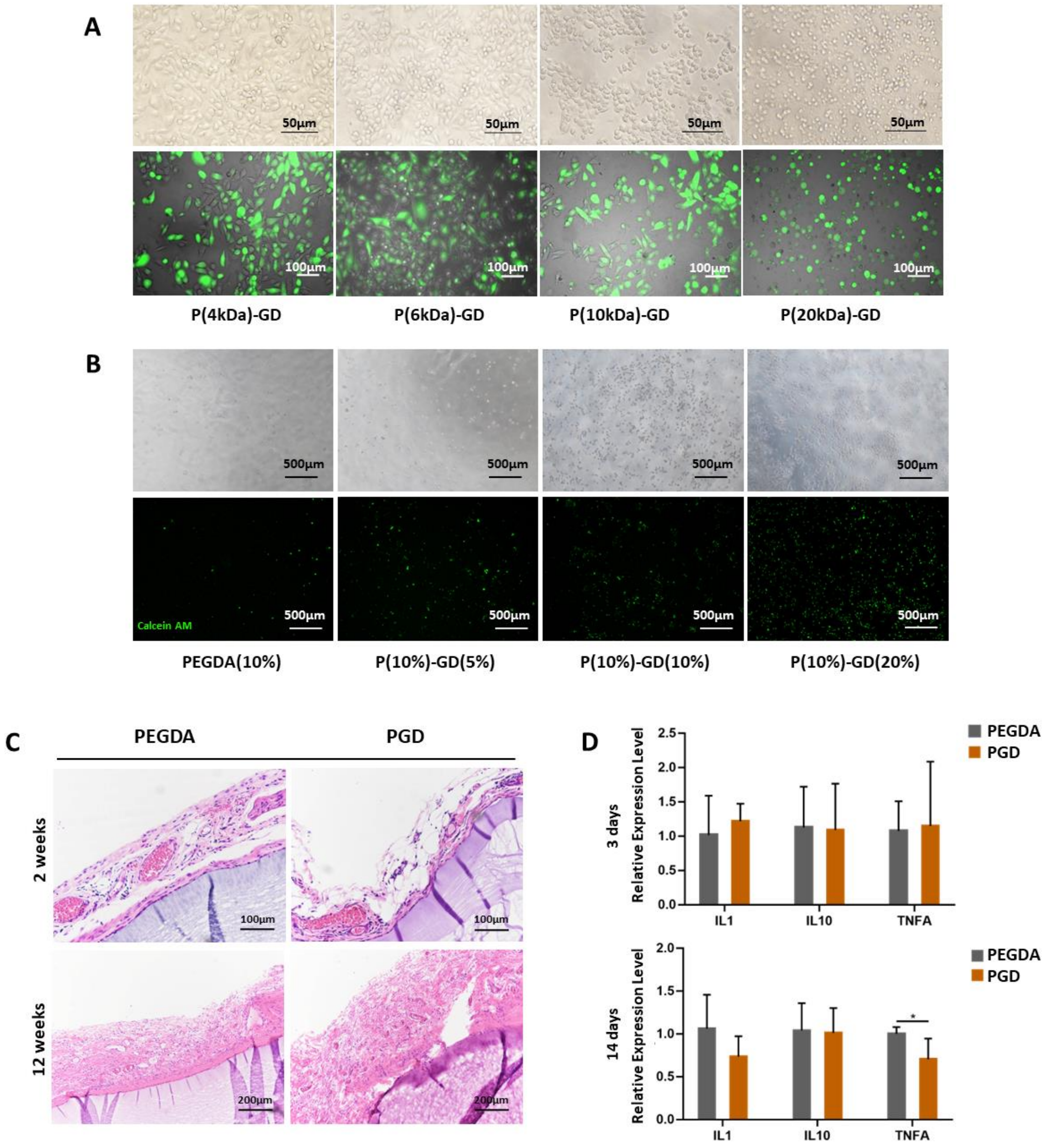

Figure S4. Human umbilical vein endothelial cells were inoculated on different PGD hydrogels to assess the biocompatibility(A)-(B); HE staining of the fibrous capsule after PGD hydrogel and PEGDA hydrogel were embedded subcutaneously in rats(C); RT-PCR analysis of inflammatory factors including IL-1, IL-10,and TNF-α after PGD hydrogel and PEGDA hydrogel were embedded subcutaneously in rats and there was no significant difference(D).

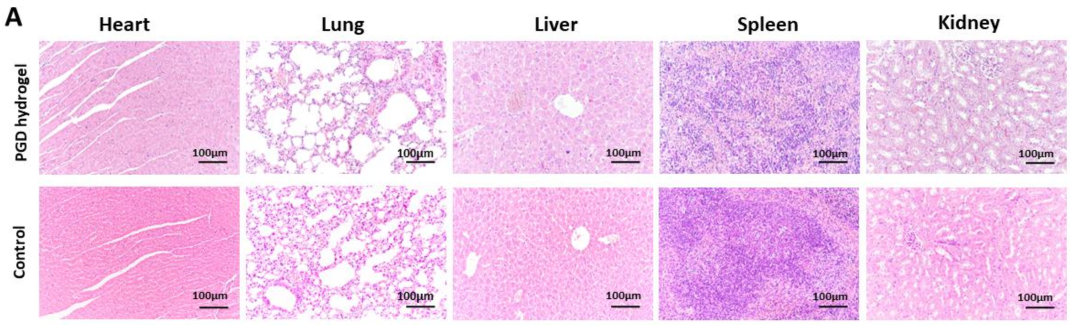

Figure S5. PGD hydrogel was implanted in the back of nude mice for 4w, and the important organs were sampled, showing no obvious abnormalities compared with the control group.

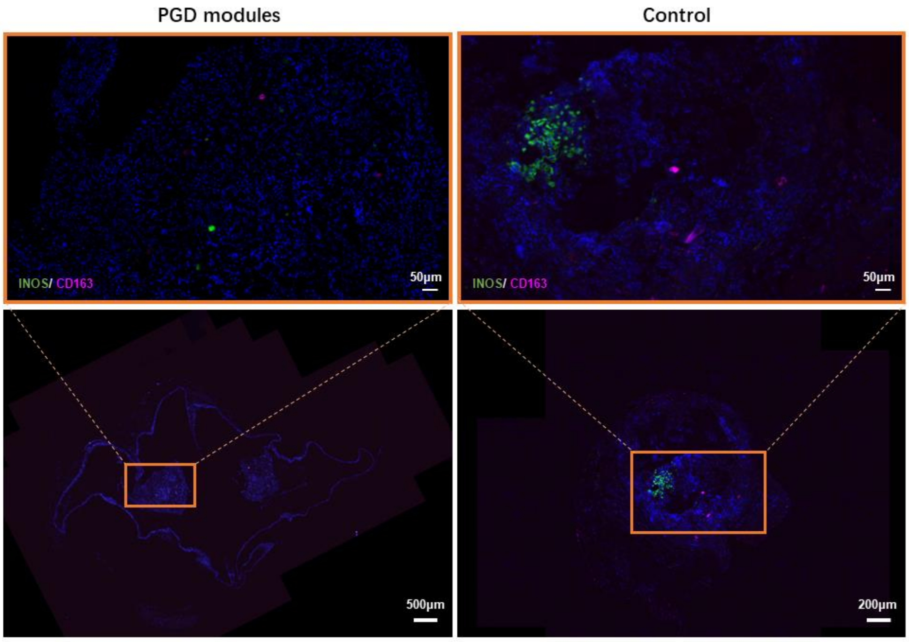

Figure S6. INOS/ CD163 double-labelling immunofluorescence staining of the tissue-engineered bone grafts in PGD hydrogel microcage modules and the control group 4 weeks after in vivo transplantation.
